# Serum markers improve current prediction of metastasis development in early‐stage melanoma patients: a machine learning‐based study

**DOI:** 10.1002/1878-0261.12732

**Published:** 2020-06-24

**Authors:** Filippo Mancuso, Sergio Lage, Javier Rasero, José Luis Díaz‐Ramón, Aintzane Apraiz, Gorka Pérez‐Yarza, Pilar Ariadna Ezkurra, Cristina Penas, Ana Sánchez‐Diez, María Dolores García‐Vazquez, Jesús Gardeazabal, Rosa Izu, Karmele Mujika, Jesús Cortés, Aintzane Asumendi, María Dolores Boyano

**Affiliations:** ^1^ Department of Cell Biology and Histology Faculty of Medicine and Nursing UPV/EHU Leioa Spain; ^2^ Biocruces Bizkaia Health Research Institute Barakaldo Spain; ^3^ Department of Psychology Carnegie Mellon University Pittsburgh PA USA; ^4^ Department of Dermatology Cruces University Hospital Barakaldo Spain; ^5^ Department of Dermatology Basurto University Hospital Bilbao Spain; ^6^ Department of Medical Oncology Onkologikoa Hospital Donostia Spain; ^7^ Biodonostia Institute Donostia Spain

**Keywords:** dermcidin, interleukins, melanoma, prognosis, serum biomarkers

## Abstract

Metastasis development represents an important threat for melanoma patients, even when diagnosed at early stages and upon removal of the primary tumor. In this scenario, determination of prognostic biomarkers would be of great interest. Serum contains information about the general status of the organism and therefore represents a valuable source for biomarkers. Thus, we aimed to define serological biomarkers that could be used along with clinical and histopathological features of the disease to predict metastatic events on the early‐stage population of patients. We previously demonstrated that in stage II melanoma patients, serum levels of dermcidin (DCD) were associated with metastatic progression. Based on the relevance of the immune response on the cancer progression and the recent association of DCD with local and systemic immune response against cancer cells, serum DCD was analyzed in a new cohort of patients along with interleukin 4 (IL‐4), IL‐6, IL‐10, IL‐17A, interferon γ (IFN‐γ), transforming growth factor‐β (TGF‐ β), and granulocyte–macrophage colony‐stimulating factor (GM‐CSF). We initially recruited 448 melanoma patients, 323 of whom were diagnosed as stages I‐II according to AJCC. Levels of selected cytokines were determined by ELISA and Luminex, and obtained data were analyzed employing machine learning and Kaplan–Meier techniques to define an algorithm capable of accurately classifying early‐stage melanoma patients with a high and low risk of developing metastasis. The results show that in early‐stage melanoma patients, serum levels of the cytokines IL‐4, GM‐CSF, and DCD together with the Breslow thickness are those that best predict melanoma metastasis. Moreover, resulting algorithm represents a new tool to discriminate subjects with good prognosis from those with high risk for a future metastasis.

## Graphical abstract

Melanoma displays a remarkable capacity for dissemination even when detected at early stages. We developed a decision rule that considers Breslow thickness of the removed malignant lesion and IL‐4, GM‐CSF, and DCD serum levels in order to foresee the risk for future metastasis development of those stage I‐II patients. This algorithm may represent a tool to design more personalized follow‐up strategies.

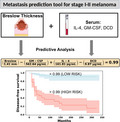

**Abbreviations**
DCD: dermcidin
GM‐CSF: granulocyte–macrophage colony‐stimulating factor
IFN: interferon
IL: Interleukin
TGF: transforming growth factor
Th: T helper

## 1 Introduction

Early and accurate classification of patients is the cornerstone of precision medicine, intimately linked to the optimal management of cancer. This is especially relevant for melanoma, the most deadly type of skin cancer due to its high metastatic capacity and the limited, although promising, therapeutic tools available to combat the advanced disease (Eggermont *et al*., 2018). Current data indicate an overall survival rate at 5 years of approximately 90% for early‐stage (stage I and II) melanomas (Bajaj *et al*., 2020; Gershenwald *et al*., 2017) and an overall survival rate at 3 years of around 55% for patients with unresectable advanced melanoma (Wolchok *et al*., 2017). In addition to the stage‐dependent severe drop in the disease‐associated survival, recurrence of the disease represents a major problem in melanoma, as more than the 10% of patients diagnosed with stage I–II melanoma have a relapse in the 5 years after the initial diagnosis (Bajaj *et al*., 2020; Lyth, 2018; von Schuckmann *et al*., 2019). Despite all the efforts to devise prevention and detection strategies, the incidence of melanoma is expected to increase in the forthcoming years (Whiteman *et al*., 2016) further supporting the benefits to be gained by investing in the development of predictive tools.

The prognosis of melanoma is currently assigned almost entirely on the basis of a limited set of histopathological markers (Kashani‐Sabet, 2014; Kashani‐Sabet *et al*., 2017). In this context, tumor thickness is the most important histopathological characteristic included in the AJCC staging system and it is officially considered as a prognostic factor for melanoma progression in clinical practice (Foth *et al*., 2016; Stiegel *et al*, 2018). However, due to the clinical and biological heterogeneity of primary melanoma, survival can vary widely even among individuals considered to be within the same stage (Elmore *et al*., 2018; Gershenwald *et al*., 2017), highlighting the need for new prognostic tools to improve the management of primary melanoma patients (Weiss *et al*., 2015). Precision medicine focuses on classifying early‐stage melanoma patients on the basis of genetic and other biochemical features in order to identify profiles that are most likely to develop into more advanced disease stages and to define more effective treatments for the metastatic disease (Gogas *et al*., 2009).

Serum is a highly accessible and valuable source of biomarkers, containing tumor and host‐related factors that are correlated with tumor behavior and patient prognosis (Palmer *et al*., 2011). Cytokines are key mediators of the immune system with either pro‐inflammatory or anti‐inflammatory activity, and they are serum factors with potential value as biomarkers. In fact, cytokine profiling is providing valuable data regarding patient classification in a wide range of diseases, including cancer (D'Angelo *et al*., 2018; Johdi *et al*., 2017; Obraztsov *et al*, 2019). In terms of tumor activity, elevated Th2 cytokines [interleukin‐4 (IL‐4), IL‐5, and IL‐13)] and decreased Th1 cytokines (IL‐2 and interferon γ (IFN‐γ)—interferon‐γ) suppress effective spontaneous antitumor immunity (Boyano *et al*., 1997; Boyano *et al*., 2000; Nevala *et al*., 2009). In addition, the IL‐17A pro‐inflammatory cytokine has been associated with poor prognosis in some tumors (Ma *et al*., 2017) and elevated levels of mainly immunosuppressive IL‐10 and transforming growth factor beta (TGF‐β) have been also correlated to bad prognosis (Lin *et al*., 2015; Zhao *et al*., 2015). Granulocyte–macrophage colony‐stimulating factor (GM‐CSF) is a hematopoietic growth factor that fulfills a fundamental role in macrophage and granulocyte differentiation. While classically linked to antitumor activities (Bhattacharya *et al*., 2015), there is growing evidence that GM‐CSF can also promote tumor progression (Reggiani *et al*., 2017; Singel and Segal, 2016; Wang *et al*., 2017a), supporting its inclusion in biomarker studies.

In a previous study carried out on a large group of melanoma patients and based on serum proteomic analysis and immunoassays, we established prognostic value of serum dermcidin (DCD) for stage II melanoma patients (Ortega‐Martínez *et al*., 2016). DCD is considered to play an important role in the cutaneous microenvironment due to its antimicrobial activity (Zeth and Sancho‐Vaello, 2017). Nevertheless, DCD is not just an antimicrobial peptide as it can stimulate keratinocytes to produce cytokines through G protein and mitogen‐activated protein kinase activation (Paulmann *et al*., 2012). These data suggest a possible relationship between *in situ* and systemic immune responses.

Accordingly, the purpose of this study was to develop a tool with clinical applications to improve the prognostic prediction of patients diagnosed with early‐stage (stage I–II) melanoma. To achieve this, we adopted a machine learning approach that incorporated the serum measurements of GM‐CSF, interferon γ (IFN‐γ), TGF‐β1, IL‐4, IL‐6, IL‐10, IL‐17A, and DCD, in conjunction with clinical–pathological features of such melanoma patients to determine the prognostic value of these parameters.

## 2 Materials and methods

### 2.1 Patients

Melanoma patients were recruited at the Dermatology Units at the Basurto and Cruces University Hospitals between 1990 and 2016. Inclusion criteria were as follows: (a) a histologically confirmed diagnosis of malignant melanoma; (b) no treatment except primary surgery (including wide local excision); and (c) no infection as judged by clinical evaluation and absence of increased infectious parameters in the blood.

Biopsies of suspicious lesions were analyzed by a melanoma pathologist. Those patients with a positive result for melanoma underwent a second surgery for a wide local excision. Patients diagnosed with stage III or IV melanoma were referred to the Oncology Unit, while stage I or II patients (from now on ‘early‐stage melanomas’) remained under the supervision of the Dermatology Unit. Upon removal of the primary tumor, clinical checkups of patients with early‐stage melanomas were scheduled every 3 months for the first 2 years of the follow‐up and every 6 months thereafter, until a 5‐year follow‐up had been completed. Annual revisions were then scheduled up to the 10th year postsurgery. The patients who developed metastasis during the follow‐up period were again examined every 3 months for 2 years after metastasis had been diagnosed. The presence or absence of metastasis was assessed in all patients by physical examination, as well as through laboratory and radiological testing (X‐rays and/or computed tomography scanning). Some patients underwent sentinel lymph node biopsy although it was not a generalized procedure.

Disease stages were classified according to the AJCC 8th edition (Gershenwald *et al*., 2017). The clinical and diagnostic data for each patient were collected retrospectively from centralized electronic and/or paper medical records. For the statistical prediction analysis, only melanoma patients at early disease stages (I and II) were included; those patients that did not develop metastasis within 2 years of follow‐up were included in the group named as ‘disease‐free’.

The study was conducted in accordance with the Declaration of Helsinki principles. It was approved by the Euskadi Ethics Committee (reference 16‐99), and written informed consent was obtained from all the subjects. The serum samples collected were stored at −80 °C at the Basque Biobank until use (https://www.biobancovasco.org/).

### 2.2 Serum samples

Venous blood samples were drawn 1 month after surgical excision of the lesions, and these samples were used to obtain serum following the protocol established at the Basque Biobank for Research. Briefly, blood samples were allowed to clot at room temperature for at least 30 min and then centrifuged at 1000 ***g*** for 10 min. The serum was collected and subsequently divided into 500 µL aliquots; aliquots were stored at −80 °C until use.

### 2.3 Quantification of Granulocyte–Macrophage Colony‐Stimulating Factor (GM‐CSF), Interferon‐γ (interferon γ (IFN‐γ)), Transforming Growth Factor beta (TGF‐β1), and Interleukins (IL) 4, 6, 10, and 17A in serum

Upon reception from the Basque Biobank, serum samples were divided into 25 μL aliquots to avoid multiple freeze/thaw cycles, and GM‐CSF, interferon γ (IFN‐γ), IL‐4, IL‐6, IL‐10, IL‐17A, and TGF‐β1 were measured using magnetic bead‐based multiple immunoassays (MILLIPLEX® MAP Kit, Human High Sensitivity T Cell Magnetic Bead Panel; EMD Millipore Corporation, Darmstadt, Germany). Each assay included two calibration curves for each of the proteins to be measured (calibration ranges: GM‐CSF, 1.22–5000 pg·mL^−1^; interferon γ (IFN‐γ), 0.61–2500 pg·mL^−1^; IL‐4, 1.83–7500 pg·mL^−1^; IL‐6, 0.18–750 pg·mL^−1^; IL‐10, 1.46–6000 pg·mL^−1^; IL‐17A, 0.73–3000 pg·mL^−1^; and TGF‐β1, 9.8–10 000 ng·mL^−1^), with eight calibration points in each curve. Two low‐ and two high‐quality controls were also included in the assays. In the case of TGF‐β1, serum samples were treated with 1N HCl, diluting the samples 1 : 4 and then adding 2 µL of 1.0 N HCl before incubating the mixture for 1 h at room temperature. The samples were then further diluted 1 : 6 in assay buffer to achieve a final dilution of 1 : 30. In the assays, we followed the protocol established by the manufacturer. The plates were read on a Luminex 100™ apparatus (Luminex Corporation, Austin, TX, USA): 50 events per bead; 150 µL of sample (or 100 µL in the case of TGF‐β1); gate settings from 8000 to 15 000; reported gain as default; and time out 100 s. The serum concentration of each protein was calculated through a 5‐parameter logistic curve‐fitting method using the xponent® software (Luminex Corporation).

### 2.4 DCD Quantification in serum

Serum DCD was measured with an ELISA Kit (Cusabio Biotech Co., Ltd, Houston, TX, USA) according to manufacturer instructions as previously described (Ortega‐Martínez *et al*., 2016).

The optical density was determined on a microplate reader (Synergy HT; BioTek Instruments, Inc., Winooski, VT, USA) set to 450 and 540 nm. Readings at 540 nm were subtracted from those obtained at 450 nm to correct for optical imperfections in the plate, and the serum DCD levels were calculated using the gen5 software (2005; BioTek Instruments, Inc.) with a 4‐parameter logistic curve fitting.

### 2.5 Statistical analysis

Variables of interest clearly deviated from a normal distribution as assessed both visually and by means of a Shapiro–Wilk test. As a consequence, all descriptive statistic was expressed as the median along with the 95% confidence interval (CI), computed by bootstrap resampling in which 10 000 samples were extracted with replacement for each variable from the original data and calculating the 95% percentile interval. Intergroup comparisons were carried out using the Kruskal–Wallis test when more than two groups were involved and a two‐sided Mann–Whitney *U* test when only two groups were compared. In the latter case, in addition to the *P*‐values, the effect sizes were reported, measured through the absolute Cliff’s delta value (Cliff, 1993), which estimates the difference between the probability that a value from one of the groups is higher than that value from the other group, and vice versa. The *P*‐values were corrected for multiple comparisons by controlling the false discovery rate (FDR) using the Benjamini–Hochberg procedure (Benjamini and Hochberg, 1995), and those whose significance level was below the threshold of 0.05 were considered significant. Likewise, further inspection of statistical significance was addressed by means of the shift function (Wilcox, 2012) as implemented in the rogme R package (Rousselet *et al*., 2017), where deciles are compared using a Harrell–Davis estimator, levels of confidence are computed by a bootstrap estimation, and the type I error is controlled to remain around 0.05 across all the decile comparisons. Comparison among observed and expected frequencies was carried out by chi‐square test, and Fisher’s exact test was employed for pairwise comparison.

### 2.6 Machine learning analysis

A machine learning analysis was performed in order to assess the power of the data to correctly classify the prognosis of melanoma patients. *In situ* melanoma patients show an extremely good prognosis, while patients are classified at stage III or IV when metastasis is detected. Therefore, we focused on patients diagnosed at stages I and II due to the clinical relevance of early metastasis prediction, of which there were 323 subjects in our cohort. Among these patients, 244 remained disease‐free and 84 developed metastasis during the follow‐up period. The predictive power of different biomarkers was inspected in three different variable domains: The histological domain, represented by the Breslow thickness; the serum domain, which involved all the serum variables indicated above; and a multimodal domain, a conjunction of the variables from the two previous domains. Missing information was imputed by removing instances containing unknown components, which reduced the input data to 211 disease‐free and 56 metastatic samples, respectively. Subsequently, a nested cross‐validation was employed to assess both the optimization and generalization of the model. In the outer loop, a 10‐fold cross‐validation repeated five times with different randomization seeds was performed to estimate the generalization error of the model. In the inner loop, a stratified 10‐fold cross‐validation was implemented for model optimization, which involved an exhaustive grid tuning of the inner hyperparameters of a pipeline assembled by robust scaling of the data, random oversampling of the class minority, a feature selection based on the weights importance order found by a logistic ridge regression model, and the fitting of classifier used. A scheme of this workflow is shown in Fig. S1.

Classification scores were computed using a battery of five classification algorithms: logistic regression (LR) with a L2 regularization term; support vector machine with a radial basis kernel; a decision tree (DT); Gaussian naive Bayes classifier; and the K‐nearest neighbors vote algorithm. All the different hyperparameters of the mentioned classifiers and the level of shrinkage and the number of features to select were tuned by an exhaustive grid search within the inner loop. Finally, for each classifier, the balanced accuracy, which calculates the raw accuracy of each sample weighted by the inverse prevalence of its true class, the precision, recall, and F1 score were reported. In addition, a receiver operating characteristic (ROC) curve was computed, such that the area under this curve (AUC) provides a measure to evaluate the classifier quality. The classifier with the highest ROC area was finally considered the most efficient one.

All the machine learning analysis was performed using scikit‐learn, a library for machine learning written in python (Pedregosa *et al*., 2011), and Imbalanced‐learn, a Python toolbox to ‘tackle the curse of imbalanced datasets in Machine Learning’ (Lemaître *et al*., 2017).

### 2.7 Disease‐free survival analysis

Once the best algorithm and subset of biomarkers to reflect the evolution of metastasis had been found, we used this combination to fit the entire stage I/II subpopulation, allowing us to compute the ROC curve. Subsequently, the optimal cutoff point on this curve was determined using the index of union method, which corresponds to computing the value where the sensitivity and specificity are the closest to the AUC, and the absolute difference between the specificity and sensitivity is minimal (Unal, 2017). This cutoff point allows us to define a class partition criterion, which separates subjects with a high probability of developing metastasis from those with a low probability, as witnessed through a Kaplan–Meier estimator implemented in a lifelines library in Python (Davidson‐Pilon *et al*., 2018).

## 3 Results

### 3.1 Patient characteristics

A total of 448 melanoma patients were recruited (187 male, 261 female), with a median age at diagnosis of 56 years (95% CI: 54.0–60.0: Table S1). Cutaneous melanoma was most often diagnosed in patients' trunks (158 patients), followed by the lower limb (121 patients). Staging was based on the AJCC system (Gershenwald *et al*., 2017), and most patients were diagnosed as stage I or II (224 and 99 patients, respectively), while only 38 were considered to be at a stage related to metastasis, stage III (30) or IV (8). However, 119 (27%) of the 448 patients recruited developed metastasis, including those with spread disease at the moment of diagnosis and those who suffered from disease recurrence during the follow‐up. Lymph node and distant metastases were the main subtypes detected. Additional information regarding the entire cohort (*in situ* and stage I–IV patients) has been included in the Table S1.

Considering patients at AJCC stages I and II as early‐stage melanoma patients (323 patients: 318 patients with cutaneous melanoma and five with noncutaneous melanoma), sex, age, and tumor location frequencies were similar to those obtained from the whole group (Table S1). Mean follow‐up for the early‐stage melanoma cohort was of 5.5 years with a median of 3.9 years. Seventy‐four of these early‐stage melanoma patients (22.9%) developed metastasis during the first years of the follow‐up. For those early‐stage patients that developed metastasis, the mean interval from the removal of the primary tumor until the diagnosis of a metastasis was 2.8 years with a median interval of 1.8 years. By contrast, 239 patients remained disease‐free (without recurrence or metastasis); mean follow‐up of these patients was 6.3 years with a median of 4.5 years. The vast majority of patients that developed metastasis (93.2%) showed a Breslow thickness > 1 mm, and ulceration was more frequently detected on those early‐stage patients with recurrent disease (35.1% vs 10.4%; χ^2^ test *P*‐value < 0.001). Metastasis development was associated with primary tumor localization (*P* < 0.05) although we could not determine any localization with significantly higher risk for metastasis development. Interestingly enough, upper limb was revealed as a location with better prognosis (*p*_FDR_ < 0.01; Table 1).

**Table 1:** Clinicopathological characteristics of stage I‐II patient cohort. ALM, acral lentiginous melanoma; LM, lentigo maligna; LMM, lentigo maligna melanoma; m/f, males/females; NM, nodular melanoma; SSM, superficial spreading melanoma.

Characteristics	Total	Disease‐free	Metastasis
	*n* (m/f)	*n* (m/f)	*n* (m/f)
Stage			
I	224 (94/130)	204 (87/117)	20 (7/13)
II	99 (40/59)	45 (17/28)	54 (23/31)

	*n* (%)	*n* (%)	*n* (%)
Tumor location^a^			
Head/neck	46 (14.5)	33 (13.5)	13 (17.8)
Trunk	125 (39.3)	101 (41.2)	24 (32.9)
Upper limb	32 (10.1)	32 (13.1)	0 (0)
Lower limb	86 (27.0)	64 (26.1)	22 (30.1)
Hand/foot	24 (7.5)	12 (4.9)	12 (16.4)
Others	5 (1.6)	3 (1.2)	2 (2.7)
nd	5	4	1
Melanoma subtypes			
SSM	170 (61.2)	151 (67.4)	19 (35.2)
NM	58 (20.9)	38 (17.0)	20 (37.0)
LMM	18 (6.5)	17 (7.6)	1 (1.9)
LM	0 (0)	0 (0)	0 (0)
ALM	17 (6.1)	8 (3.6)	9 (16.7)
Others	15 (5.4)	10 (4.5)	5 (9.3)
nd	45	25	20
Breslow thickness (mm)			
≤ 1.0	159 (52.1)	155 (63.0)	4 (6.8)
> 1.0–2.0	71 (23.3)	53 (21.5)	18 (30.5)
> 2.0–4.0	45 (14.8)	27 (11.0)	18 (30.5)
> 4.0	30 (9.8)	11 (4.5)	19 (32.2)
nd	18	3	15
Ulceration^b^			
Yes	52 (16.1)	26 (10.4)	26 (35.1)
No	271 (83.9)	223 (89.6)	48 (64.9)
Sentinel lymph node			
Not conducted	68 (36.6)	54 (36.7)	14 (35.9)
Negative	116 (62.4)	93 (63.3)	23 (59.0)
Positive	2 (1.0)	0 (0)	2 (5.1)
nd	137	102	35

^a^ Metastasis development was associated with primary tumor localization (χ^2^ test *P*‐value < 0.05).

^b^ Ulceration was more often observed on early‐stage patients that developed metastasis (χ^2^ test *P*‐value < 0.001).

### 3.2 Analysis of serum GM‐CSF, IL‐4, IL‐6, IL‐10, IL‐17A, interferon γ (IFN‐γ), TGF‐β, and DCD

As previously mentioned, blood samples were collected upon surgical excision of melanoma lesions. In a first analysis including the entire cohort of recruited patients (*in situ* and stage I–IV melanoma patients), the amount of GM‐CSF, IL4, IL‐6, IL‐10, IL‐17A, and TGF‐β detected in serum was independent of the age of the melanoma patients and it did not vary between the sexes. However, there were significant differences between the sexes in the levels of interferon γ (IFN‐γ) and DCD (|δ| = 0.2, *p*_FDR_ < 0.01 and |δ| = 0.2, *p*_FDR_ < 0.01, respectively: data not shown). Of the proteins studied, the median serum level in the melanoma patients at the time of diagnosis was considered according to the stage of the tumor. No significant differences were observed in the median serum levels among patients of different AJCC stages (including *in situ* and stages I–IV), nor were any differences found between the distinct histological subtypes of melanoma (data not shown). Focused on early‐stage patients (stages I–II), none of the cytokines or DCD revealed statistically significant differences when comparing stage I vs stage II patients (Table 2).

**Table 2:** Comparison of serum cytokine and DCD levels between AJCC stage I and II patients who were disease‐free or developed metastasis during follow‐up. Median values of serum analysis. Serum levels of GM‐CSF, IL‐4, IL‐6, IL‐10, IL‐17A, and interferon γ (IFN‐γ) are expressed in pg·mL^−1^. TGF‐β levels are expressed in ng·mL^−1^ and DCD in µg·mL^−1^. Square brackets reflect the lower and upper 95% confidence intervals of the median. |δ| = Cliff’s delta, and *p*_FDR_ is the *P*‐value estimated by a two‐sided Mann–Whitney *U* test corrected for FDR.

	AJCC stage^a^		|δ|, *p*_FDR_	Disease progression		|δ|, *p*_FDR_
	I	II		Disease‐free	Metastasis	
GM‐CSF	122.56 [107.17–140.39]	137.86 [104.20–170.74]	< 0.01, 0.96	121.39 [103.17–138.63]	131.55 [101.01–153.24]	0.03, 0.72
IL‐4	35.95 [28.88**–**41.50]	37.81 [28.42**–**56.31]	0.10, 0.44	31.96 [27.76**–**38.06]	62.27 [39.06**–**92.21]	**0**.**30, < 0**.**01**
IL‐6	3.30 [2.91**–**3.98]	4.27 [3.23**–**5.46]	0.14, 0.44	3.29 [2.82**–**3.87]	4.71 [3.3**–**5.9]	**0**.**20, 0**.**04**
IL‐10	10.23 [7.95**–**13.76]	11.34 [7.38**–**16.35]	0.06, 0.73	11.23 [7.89**–**14.83]	10.03 [7.78**–**15.31]	0.05, 0.67
IL‐17A	19.35 [17.12**–**22.24]	16.51 [12.85**–**22.07]	0.04, 0.79	17.88 [16.33**–**20.06]	20.09 [14.89**–**24.33]	0.04, 0.67
interferon γ (IFN‐γ)	17.95 [15.92**–**20.04]	19.07 [13.94**–**24.57]	0.01, 0.96	17.23 [14.84**–**19.34]	22.40 [16.99**–**26.91]	0.12, 0.37
TGF‐β	49.18 [45.30–54.21]	46.67 [40.13–55.30]	0.11, 0.44	49.71 [45.32–53.68]	48.18 [40.13–60.44]	0.04, 0.67
DCD	4.81 [4.31**–**5.01]	4.43 [3.98**–**4.88]	0.06, 0.73	4.78 [4.39**–**5.01]	4.38 [3.92**–**4.84]	0.07, 0.67

^a^ The American Joint Committee of Cancer (AJCC) staging system for melanomas was used.

Bold values highlight cytokines (IL‐4 and IL‐6) with significant differences among early‐stage melanoma patients that developt metastasis and those that remained metastasis‐free during the follow‐up.

To analyze the prognostic value of these proteins, we compared serum cytokine and DCD levels of early‐stage melanoma patients that remained disease‐free at the end of the follow‐up period, with those obtained from early‐stage patients who developed metastasis. Significant differences were observed in the serum IL‐4 and IL‐6 levels between these two groups of patients and were associated with a moderate effect size (|δ| = 0.30 *p*_FDR_ < 0.01 and |δ| = 0.20 *p*_FDR_ = 0.04: Table 2). At the time of diagnosis, the serum IL‐4 levels of patients who developed metastasis doubled those observed in patients who remained disease‐free (62.27 vs 31.96 pg·mL^−1^, respectively, *P* < 0.01). A similar trend was detected regarding serum IL‐6 (4.71 vs 3.29 pg·mL^−1^, *P* < 0.04). No significant differences in serum GM‐CSF, IL‐10, IL‐17A, interferon γ (IFN‐γ), TGF‐β, and DCD were observed.

### 3.3 Prognostic power of the melanoma markers

**Fig. 1:**
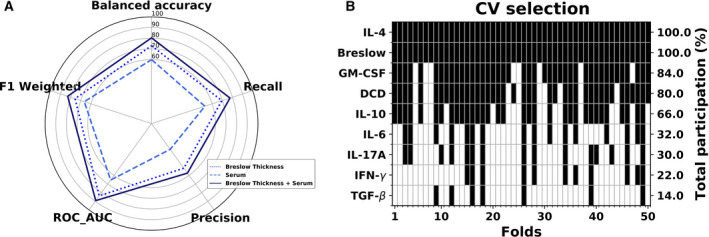
LR analysis. (A) Classification of the three variable domains considered: Breslow thickness, cytokines, and DCD serum variables. (B) In the scenario combining histological and serum variables, their participation across the folds is provided by the feature selection step in the inner cross‐validation loop. Black colors in each column denote the predictors that were included in the final LR model in each of these folds. Data are from the early‐stage melanoma cohort (*n* = 323).

The performance of the different classifiers was assessed for the subpopulation of subjects at AJCC stages I and II (Table S2). In all the three domains, a LR classifier exhibited the best performance through the ROC area, and the most generalizable results were reflected by the smallest gap between the training and test scores. The Breslow thickness represents a biomarker of melanoma metastasis that correctly classified 73% of the patients and generating 83% of the ROC area (Fig. 1A). Although the serum levels solely had a poorer prognostic value, when combined with the Breslow thickness, they significantly improved the cross‐validated performance of this biomarker, exceeding a balanced accuracy of 80% (Wilcoxon signed‐rank test, *P* < 0.01) and a ROC area close to the 90% (Wilcoxon signed‐rank test, *P* < 0.01). Furthermore, these data clearly pointed to the cytokines IL‐4, GM‐CSF, and DCD as the most powerful biomarkers in predicting melanoma metastasis in conjunction with the Breslow thickness. Indeed, these parameters were selected at least the 80% of the times in all the partitions after the feature selection process (see panel B of Fig. 1). This subset of variables was followed by the IL‐10 being 66% of the times selected and the IL‐6, IL‐17A, interferon γ (IFN‐γ), and TGF‐β below the 50%. In sum, cytokines and DCD alone represent a weak source of melanoma prognosis. For instance, the subset composed of GM‐CSF, IL‐4, and DCD submitted to the LR classifier yields a 59.82% balanced accuracy, which is only moderately better than predicting by random chance, and a 66.97% ROC area. However, their importance resides in the complementary role as to melanoma metastasis prediction when they are combined with a strong predictive biomarker such as Breslow thickness.

**Fig. 2:**
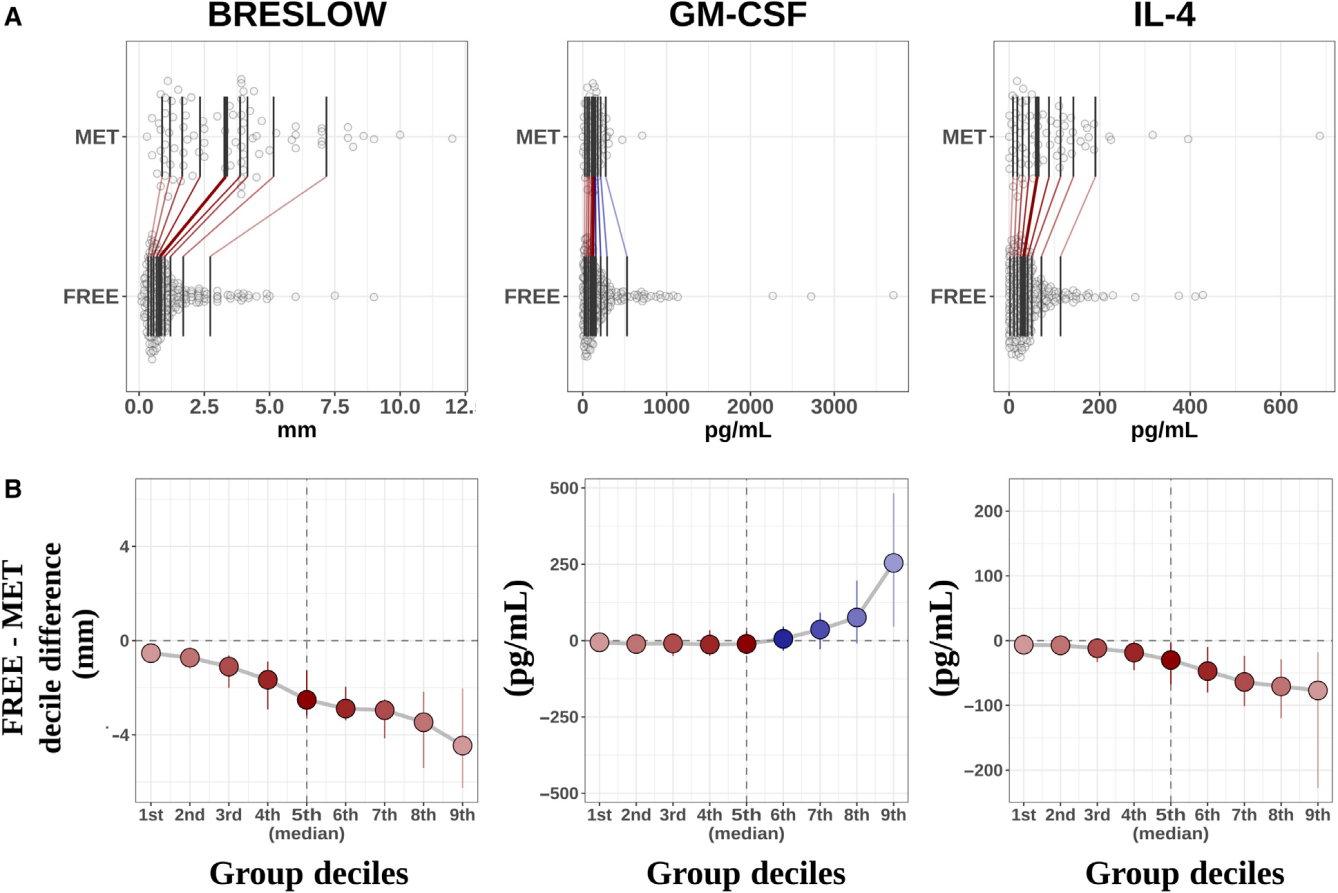
(A) Decile distribution of metastatic and disease‐free subjects for the Breslow thickness, GM‐CSF, and IL‐4. (B) For this subset of features, the shift function displays the difference between the deciles in both subgroups of subjects. Positive values of the shift function are in blue, corresponding to larger decile values in the disease‐free group than in the metastatic group, while red values illustrate the opposite scenario.

Notably, the decile distribution for these potential biomarkers exhibited a clear tendency to separate between subjects in stages I and II who developed metastasis and those who remained disease‐free (panel A in Fig. 2). Both the distribution of the serum IL‐4 levels and the Breslow thickness were higher in the metastatic subpopulation, whereas this tendency switched toward lower levels of GM‐CSF. Moreover, when the differences in the distribution of these variables were addressed by means of the shift function, their predictive power was clearly evident, especially that of the Breslow thickness where the separation of the melanoma outcome was significant across its whole spectrum. This was followed by that of IL‐4, which began to display a significant separation around the median, whereas GM‐CSF started to discriminate these subpopulations above its 8th decile (panel B in Fig. 2). For DCD, nevertheless, this class‐separating tendency is not as evident (Fig. S2), which may denote a synergetic role emerging beyond the univariate scenario. This also seems to be the same for the rest of variables of interest (Fig. S2).

**Fig. 3:**
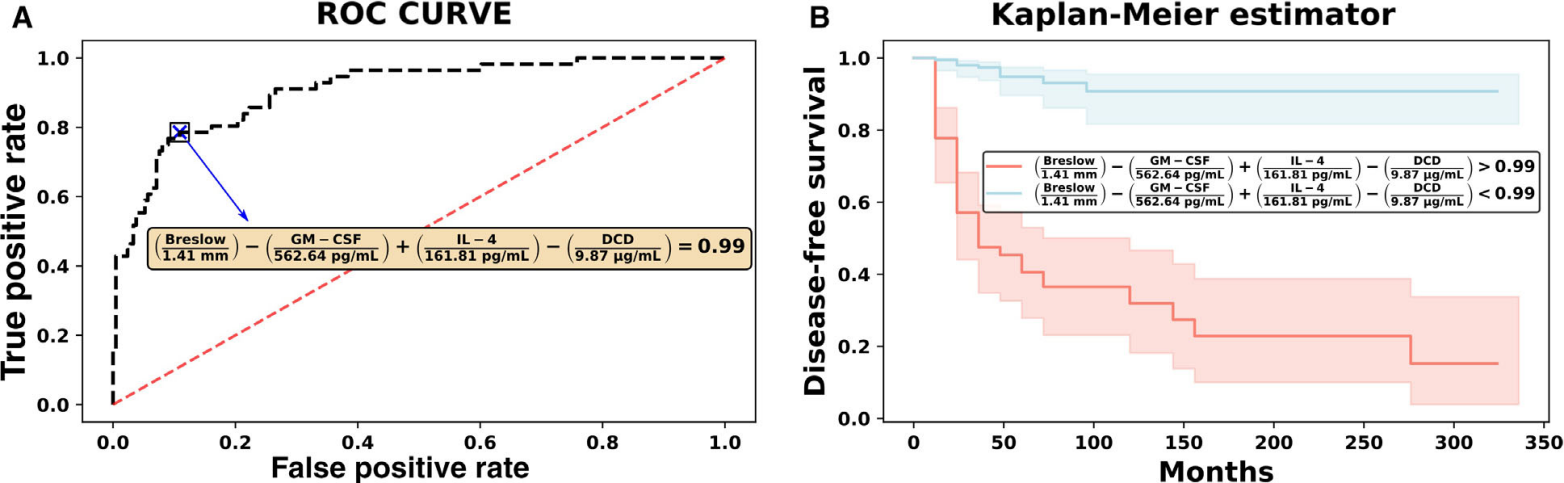
(A) ROC curve from the whole‐stage I/II dataset. The optimal cutoff point on this curve defines a plane that maximally separates metastatic and disease‐free progression. The best subset of biomarkers corresponds to Breslow thickness, IL‐4, GM‐CSF, and DCD. (B) Kaplan–Meier analysis. The cutoff plane provides a condition to significantly separate subjects with a worse prognosis from those with a better prognosis.

More importantly, these findings can be easily incorporated into clinical protocols by providing a general optimum cutoff from the data from which a prediction of metastasis can be performed. Using the same subpopulation of subjects (I/II melanoma patients), we fitted the entire data using the best classifier and the subset of features found previously (i.e., a LR classifier with the features Breslow thickness, GM‐CSF, IL‐4, and DCD), and we computed the optimal point on the ROC curve that corresponded in this case to FPR (1‐specificity) = 0.11 and TPR (sensitivity) = 0.79 (see panel A, Fig. 3). This point defines a critical threshold that allows us to separate subjects in terms of their prognosis, which for our classifier can be easily translated into a constraint as follows:

(1) $$\left(\frac{\mathrm{Breslow}}{1.41\;mm}\right)-\left(\frac{GM-CSF}{571.28\;pg\cdot mL^{-1}}\right)+\left(\frac{IL-4}{168.1\;pg\cdot mL^{-1}}\right)-\left(\frac{\mathrm{DCD}}{9.87\;\mu g\cdot mL^{-1}}\right)=0.99$$

This equation therefore defines a hyperplane in our feature space such that any subject lying above it is classified as metastatic and those below it are considered disease‐free. Furthermore, subjects stratified with respect to this critical threshold could be differentiated by their probability of eventually developing metastasis during the follow‐up period (Kaplan–Meier log‐rank test *P* < 0.001, as shown in panel B of Fig. 3).

Remarkably, we found a prognostic plane involving the serum levels of IL‐4, GM‐CSF, and DCD in conjunction with the Breslow thickness that could accurately classify subjects according to their melanoma outcome. This equation could be easily translated to a clinical setting, and inspecting the signs of the coefficients in this equation, we can clearly see that an increase in IL‐4 and the Breslow thickness tend to shift subjects above this plane, indicating a worse prognosis, whereas GM‐CSF and DCD levels act in the opposite direction.

Finally, in order to account for the effect of additional prognostic factors in the present findings, we repeated the previous analyses considering also age, sex, and ulceration as predictive variables in the full feature matrix used to train the LR. Their inclusion yields an increase in the balanced accuracy rate (81.85%), precision (60.55%), and recall (78.60%) and a slight decrease in the ROC area (88.80%). Such changes are primarily mediated by the role of gender as an important predicting factor due to the number of times it is selected during the feature selection process. Yet, it still is preceded by Breslow, IL‐4, GM‐CSF, and DCD variables. In contrast, both age and ulceration, respectively, contribute fewer times to forming the predictive model (Fig. S3). Subsequently, a Bayesian approach shows that a model incorporating age, gender, and ulceration in addition to the variables of the rule provided by the prognostic equation (1) adds such amount of complexity to the model that leads to an increase in the Bayes information criterion (BIC) from 334.39 to 355.54, providing a very strong evidence against their inclusion (Bayes Factor > 150) (Wagenmakers, 2007). Likewise, in spite of the aforementioned predictive importance of gender, its sole inclusion is also strongly disfavored (BIC = 349.07).

## 4 Discussion

An accurate diagnosis is an essential first step in cancer management. Most melanoma cases are detected at early disease stages, and when possible, excision biopsy is the selected procedure to treat suspicious melanocytic lesions. According to the AJCC 8th classification, Breslow thickness, together with the ulceration, is important variable that should be considered in tumor staging (https://cancerstaging.org/). In addition, several histological biomarkers (e.g., Melan‐A, Pmel) are routinely employed for diagnostic purposes (Mohammadpour *et al*., 2019). In this regard, important efforts are being made in order to achieve less invasive techniques or to improve the accuracy of diagnostic markers (Svedman *et al*., 2016). Indeed, early and precise prognostic markers are urgently needed for melanoma due to its strong metastatic capacity and, particularly, given the low survival rate of metastatic patients (5‐year overall survival probability of 23.4–32% for stage IIID patients; Bajaj *et al*., 2020; Gershenwald *et al*., 2017). Moreover, risk of recurrence for early‐stage (stage I–II) melanomas must also be considered with 5‐year probability values ranging from 8.8% to 74.5% depending on the substage (Bajaj *et al*., 2020). According to Rutkowski and Lugowska (2014), highest recurrence rate is observed within 2–3 years after surgical treatment while recurrence probability decreases to < 5% in patients with treated stage I–III melanomas and 5 years of disease‐free follow‐up. In a cohort similar to ours, median time until recurrence was set at 1.7 years although with clear differences among tumors with different Breslow thickness (Lyth, 2018). Our data, with a median time of 1.8 years, corroborate published data. According to these figures, intense medical monitoring should be implemented in the first 2–3 years after treatment, even for early‐stage cases, representing an important medical and economic burden. Therefore, it would be useful if early patients could be rapidly classified into high or low recurrence risk groups when contemplating efficient and sustainable personalized follow‐up programs. In this sense, to assuage the unpredictable clinical behavior of melanoma, much research has focused on the discovery of prognostic factors to improve the prognostic accuracy for this type of skin cancer (Kashani‐Sabet, *et al*., 2017). In this regard, our study focused on the discovery of prognostic biomarkers capable of evaluating the metastatic risk of patients identified at early stages of the disease (stages I–II). Moreover, we defined a clinically applicable mathematical tool to accurately classify such melanoma patients. Ideally, analyzed factor should have been able to accurately predict metastatic risk of all stage I and II substages as it is well known that despite being considered early stages, differences are on the recurrence‐free survival probabilities of different substages (Bajaj *et al*., 2020; Gershenwald *et al*., 2017; von Schuckmann *et al*., 2019). Nevertheless, our cohort size did not allow such stratification. In addition, it is described that melanoma‐specific survival rate describes a continuous decrease over time even for stage I melanoma patients, classically linked to very good prognosis (Lo *et al*., 2018). In fact, among stage I patients that die due to melanoma, only 29% of deaths occur during the first 5 years after diagnosis. Therefore, with a median follow‐up period of 4.5 years, we may have not been able to detect late recurrences although it represents a powerful tool to correctly classify the major part of early‐stage patients with high risk for metastasis development.

Currently, predicting patient outcome mainly relies on staging based on the histopathological parameters described previously, while treatment options are often based on the BRAF mutation (Karagiannis *et al*., 2015). Nevertheless, patient monitoring, especially upon surgical removal of the primary tumor, requires other variables to be analyzed. As a systemic system for information transfer, serum represents a complex but accessible sensor. To date, LDH has been one and perhaps the only clinical serological biomarker for melanoma, with increasing values interpreted as disease progression. However, an increase in serum LDH levels may also occur in other settings, which means employing some caution before reaching any conclusion (Karagiannis *et al*., 2015).

Serological cytokines reflect the general immunological state of the body, offering information regarding the cytokines released by tumors and those that accumulate in the tumor microenvironment (Wang *et al*., 2017b). The melanoma microenvironment contains stromal cells and immune cells like T or B lymphocytes, NK cells, or tumor‐associated macrophages (Jiang *et al*., 2019; Terren *et al*., 2019). Most of these cells secrete cytokines that may play a key role in inhibiting or promoting tumor progression (Jiang *et al*., 2019). The pro‐inflammatory cytokines IL‐4 and IL‐6, produced either by host immune cells or by tumor cells themselves, are associated with tumor malignancy in patients and animal cancer models (Ito *et al*., 2017; Setrerrahmane and Xu, 2017; Surcel *et al*., 2017). At the cutaneous level, keratinocytes secrete IL‐6 in order to enhance T‐cell‐mediated antitumor activity, and therefore, high IL‐6 levels are considered a marker for immune system upregulation (Setrerrahmane and Xu, 2017; Surcel *et al*., 2017). IL‐4 is the most important Th2 cytokine, and it is mainly produced by activated T cells, mast cells, basophils, and eosinophils in order to regulate lymphocyte proliferation and survival (Setrerrahmane and Xu, 2017). Interestingly, elevated serum IL‐6 was correlated with a poor prognosis in melanoma, while IL‐4 is thought to promote the proliferation and survival of several cancer cells (Gocheva *et al*., 2010; Jobe *et al*., 2018; Yu *et al*., 2019). In line with previous findings, early‐stage (I or II) melanoma patients that developed metastasis had significantly higher levels of serum IL‐4 and IL‐6 than patients who did not develop metastasis during the follow‐up.

Our previous attempt to identify novel serological prognostic markers identified a threshold for serological DCD that was associated with a poor prognosis value for melanoma patients diagnosed specifically at AJCC stage II (Ortega‐Martínez *et al*., 2016). Consistent with this finding, DCD, a major human antimicrobial peptide in human skin (Paulmann *et al*., 2012; Zeth and Sancho‐Vaello, 2017), was also recently proposed as a serological marker for the diagnosis and staging of hepatocellular carcinoma (Qiu *et al*., 2018).

The current study including a new cohort of melanoma patients revealed that DCD is a marker of metastatic progression, although other serological parameters appear to have greater predictive potential than DCD, such as IL‐4 and GM‐CSF. These differences with our previous study (Ortega‐Martínez *et al*., 2016) may be due to the patient stratification, as both stage I and stage II melanoma patients were included in separate groups for the DT analysis.

It is well described that serum components may vary before, during, and after tumor excision surgery (Grimm *et al*., 2016; Kahn *et al*., 2015) which supports the need for a standardized protocol for blood collection. In this study, blood was drawn with a controlled protocol, always 1 month after tumor excision. The fact that blood was withdrawn after surgery may have diminished the detection of tumor‐related cytokines; nevertheless, it represents an excellent sensor for the detection of melanoma‐related effects even when tumor mass remains undetectable.

The Breslow thickness is a crucial prognostic factor, with substantial evidence confirming a direct relationship between Breslow thickness and survival (Stiegel *et al*., 2018). Accordingly, we show that Breslow thickness is the most important risk factor for the malignant progression of melanoma as this variable achieved highest predictive scores in our analysis. Nonetheless, a significant increase in the predictive power of Breslow thickness was achieved by combining it with data regarding serum IL‐4, GM‐CSF, and DCD, resulting in the development of an algorithm to identify early‐stage melanoma patients with a high risk of developing metastasis during the follow‐up. According to this algorithm, a high Breslow thickness and serum IL‐4 levels in early‐stage melanoma patients are associated with a poor prognosis, whereas GM‐CSF and DCD levels decrease in patients in whom the disease outcome is poor. These results are consistent with other studies describing an antitumor effect of GM‐CSF and DCD (Hong, 2016; Ortega‐Martínez *et al*., 2016). Our data also revealed the importance of IL‐10 and IL‐6 in predicting metastatic progression, albeit they provide a subleading and fluctuating contribution to melanoma outcome prediction compared to Breslow thickness and serum levels of IL‐4, GM‐CSF, and DCD.

The presence of ulceration is another important factor that has been usually associated with a lower survival rate (Gershenwald *et al*., 2017). It also increases the aggressiveness of melanomas and leads thin tumors to exhibit comparable survival rates to those nonulcerated at later stages (Hawkins *et al*., 2019). Interestingly, our data display a significantly greater proportion of metastatic cases in patients with ulcerated tumors compared to those cases lacking of ulceration. However, this is not translated into an improvement in the classification rates for melanoma outcome when combined with the rest of the biomarkers considered in this study. Since both ulceration and Breslow thickness show a significantly large correlation (Point‐Biserial *r* = 0.51, *P*‐value < 0.001), very little to null information is gained when both variables are combined in the same predictive model.

According to the 8th edition of the AJCC (Gershenwald *et al*., 2017), SLNB should be routinely applied as a staging procedure for patients with T1b‐T4. As shown by von Schuckmann *et al*. (2019), SLNB provides a more accurate classification of patients and a consequent increase rate of disease‐free survival for I–II stage patients. Nevertheless, our results, although limited, are independent of whether SLNB was applied or not. Indeed, similar disease‐free vs metastasis ratios were observed on those individuals recruited prior to the implantation of the SLNB procedure compared with those subjected to SLNB. Of mention, we obtained SLNB‐related information only for the 57% of all stage I‐II patients as this procedure was not standardized for the entire period of patient recruitment. As a consequence, the insertion of such variable in the predictive model would substantially reduce the size of the dataset, whereby increasing the risk of overfitting. Furthermore, and in agreement with patient classification in stages I–II, the results of those who underwent SLNB were negative, so no information gain across individuals would be achieved by its inclusion in this particular scenario. Nonetheless, we consider that incorporation of the information regarding SLNB would be definitely relevant when dealing with patients across all melanoma stages. Future studies incorporating SLNB detection in the patients’ clinical routine will attempt to explore the influence of this variable in the present findings.

In summary, the use of machine learning techniques has helped to define an algorithm capable of accurately classifying early‐stage melanoma patients with a high or low risk of developing metastasis. The equation generated took into account the serum IL‐4, GM‐CSF, and DCD levels and the Breslow index, and it could stratify melanoma patients to be triaged at the time of diagnosis and initial surgery, or it could also be used clinically to determine whether stage I or II melanoma patients should receive adjuvant therapy to prevent metastatic progression. In addition, our findings are valid regardless of the melanoma location and histology. Future studies will attempt to validate current data and specify the predictive rules in more stratified scenarios, for which the recruitment of larger populations of patients than in the current study will be required to overcome the chance of data overfitting.

## 5 Conclusions

We have developed a prognostic equation that considers the serum IL‐4, GM‐CSF, and DCD levels, along with the Breslow thickness to accurately classify melanoma outcome in early‐stage (I‐II) patients. In this sense, a rigorous follow‐up is recommended for early‐stage melanoma patients with a high Breslow thickness, high serum IL‐4 levels, and low GM‐CSF and DCD levels at the time of diagnosis, given the elevated risk for these patients to develop metastasis during follow‐up.

## Conflict of interest

The authors declare no conflict of interest.

## Author contributions

MDB and AAs carried out the project administration; conceived the study; supervised the work; wrote, reviewed, and edited the original draft; and acquired funding. FM, SL, and MDGV performed the serological examinations and methodology. JR and JMC have been the major contributors in statistical analysis, machine learning, and survival analysis. JLDR, ASD, JG, and RI as specialized dermatologists have diagnosed and done the follow‐up of all the patients included in this study. Moreover, they have analyzed and interpreted the data according to the clinical significance of the results. AAp and GPY analyzed and interpreted all the clinical and experimental data, and they have contributed to writing, review, and editing of the manuscript. PAE and CP contributed to sample curation, revision of clinical data, and tumor evolution of patients. All the authors read and approved the final manuscript. FM, SL, and JR contributed equally to this work.

## Supporting information


**Fig**.** S1**. Workflow of the machine learning analysis.

**Fig**.** S2**. Shift function for the rest of variables of interest, displaying the difference between the deciles of the subgroup of disease‐free and metastatic subjects. Positive values of the shift function are in blue, corresponding to larger decile values in the disease‐free group than in the metastatic group, while red values illustrate the opposite scenario.

**Fig**.** S3**. Participation across the folds provided by the feature selection step in the inner cross‐validation loop when confounding variables age, sex and ulceration are incorporated. Black colors in each column denote the predictors that were included in the final logistic regression model in each of these folds. Data from the early‐stage melanoma cohort (*n* = 323).

**Table S1**. Patient characterization: whole recruited cohort

**Table S2**. Train and test prediction scores in the three domains of variables for the battery of algorithms used.

## Data Availability

The datasets generated during and/or analyzed during the current study are not publicly available to avoid their potential misuse or misinterpretation, but they are available from the corresponding author on reasonable request. The code used for the analysis and plots is available in https://github.com/jrasero/citosines‐melanoma. The manuscript has been preprinted in bioRxiv server https://doi.org/10.1101/632455.
