# Supplementary figures and images for: Serum markers improve current prediction of metastasis development in early‐stage melanoma patients: a machine learning‐based study
Source: Mol Oncol. 2020 Jun 24;14(8):1705–18. doi: 10.1002/1878-0261.12732 (PMC7400797; doi:10.1002/1878-0261.12732)

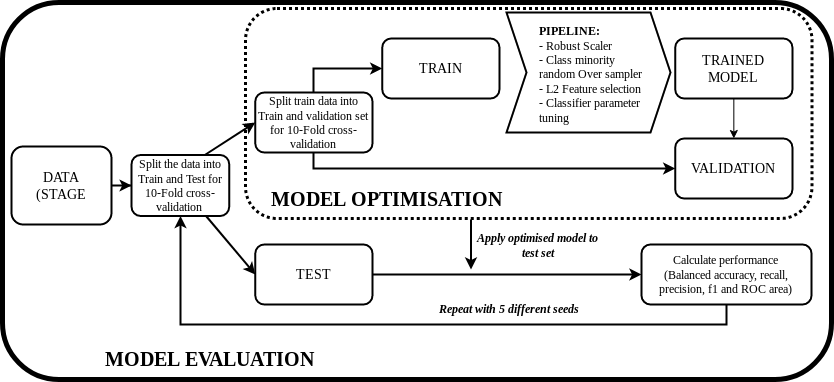

Supplement: Supplementary file 1 — Fig. S1. Workflow of the machine learning analysis. [file MOL2-14-1705-s001.tiff]

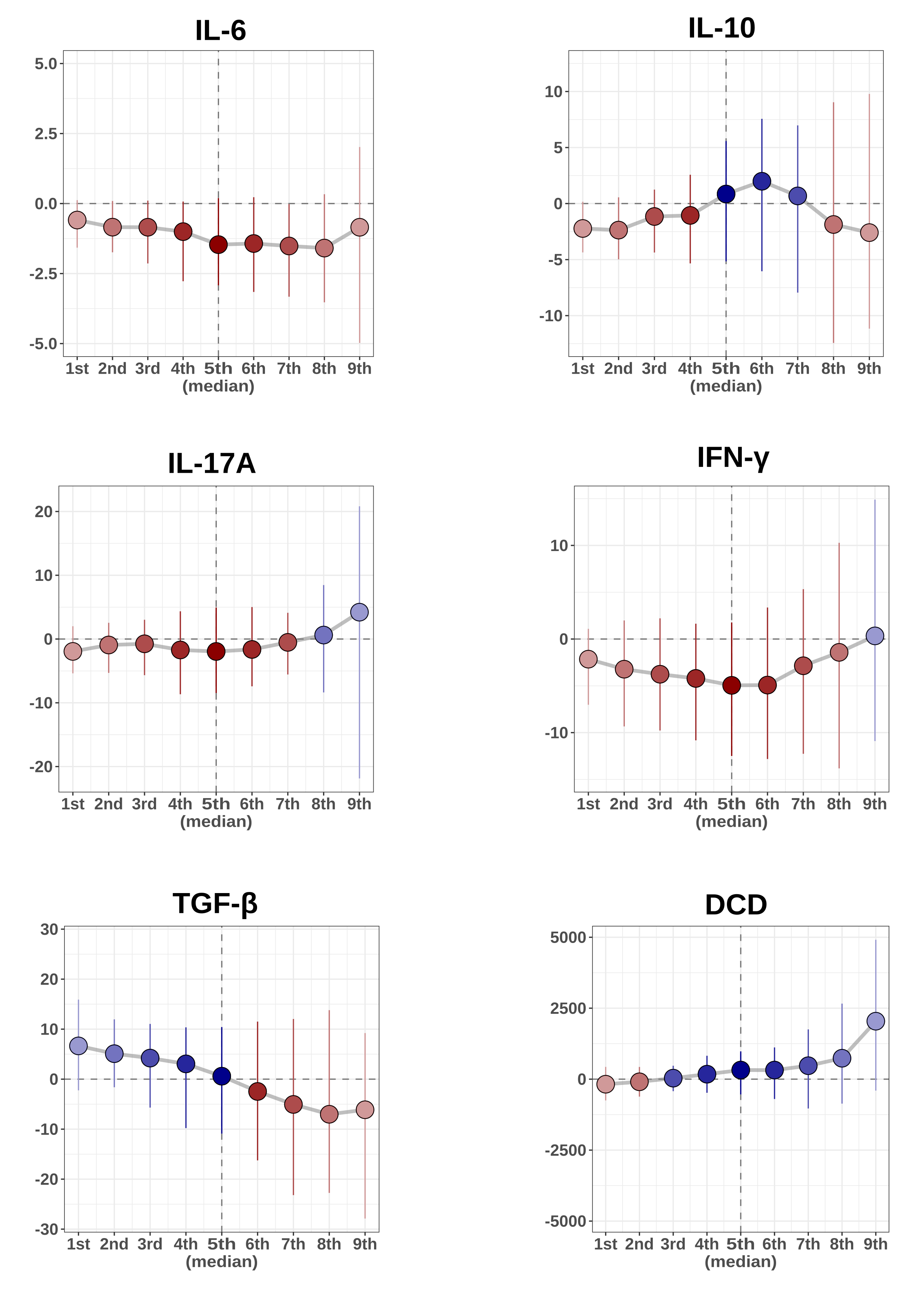

Supplement: Supplementary file 2 — Fig. S2. Shift function for the rest of variables of interest, displaying the difference between the deciles of the subgroup of disease‐free and metastatic subjects. Positive values of the shift function are in blue, corresponding to larger decile values in the disease‐free group than in the metastatic group, while red values illustrate the opposite scenario. [file MOL2-14-1705-s002.tiff]

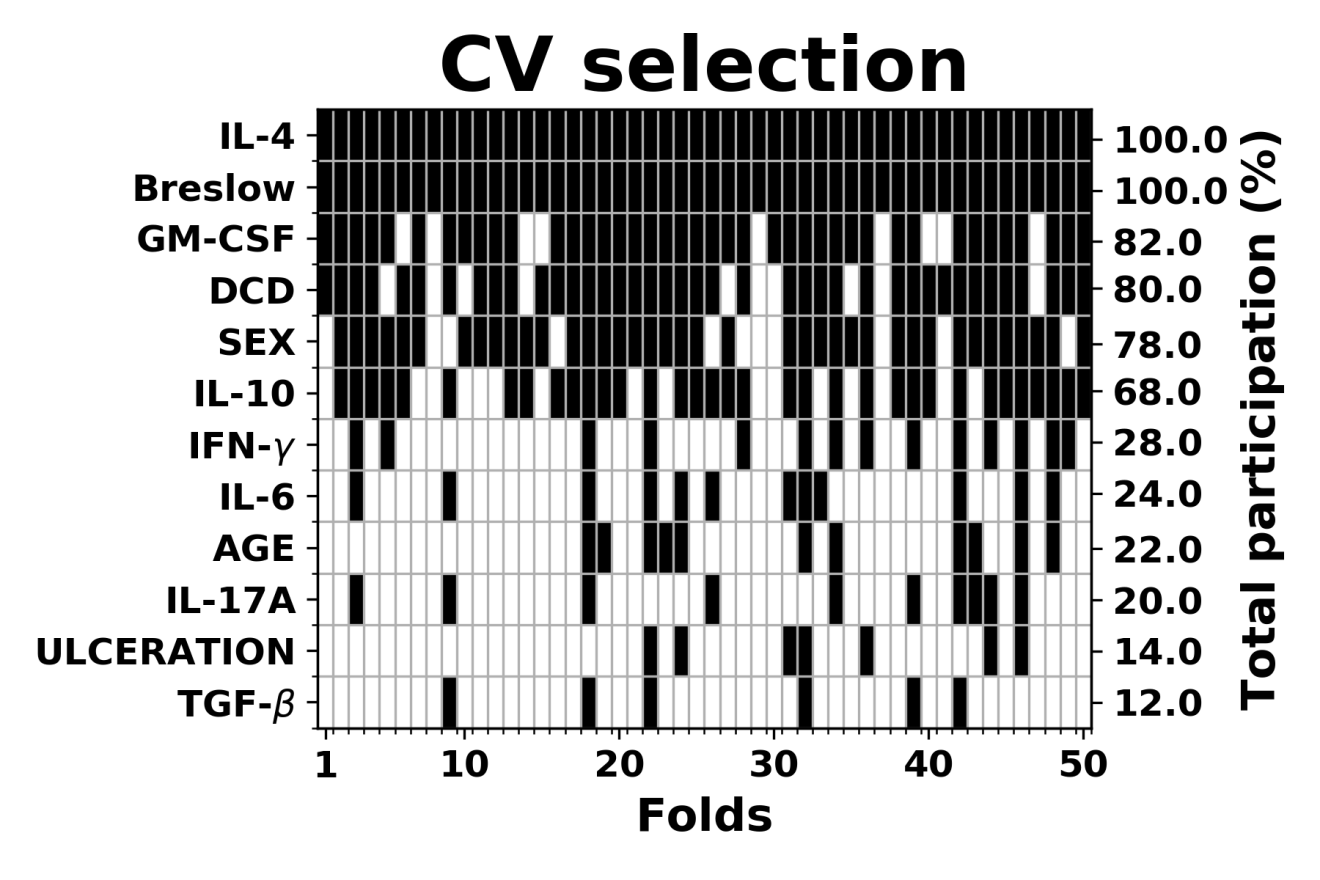

Supplement: Supplementary file 3 — Fig. S3. Participation across the folds provided by the feature selection step in the inner cross‐validation loop when confounding variables age, sex and ulceration are incorporated. Black colors in each column denote the predictors that were included in the final logistic regression model in each of these folds. Data from the early‐stage melanoma cohort (n = 323). [file MOL2-14-1705-s003.tif]
