# Supplementary material for: Serum markers improve current prediction of metastasis development in early‐stage melanoma patients: a machine learning‐based study
Source: Mol Oncol. 2020 Jun 24;14(8):1705–18. doi: 10.1002/1878-0261.12732 (PMC7400797; doi:10.1002/1878-0261.12732)
Supplement: Supplementary file 4 — Table S1. Patient characterization: whole recruited cohort [file MOL2-14-1705-s004.docx]

**Table S1. Patient characterization: whole recruited cohort.**

| **Characteristics** | **AJCC^1^** | | | | |
| --- | --- | --- | --- | --- | --- |
|  | **in situ** | **I** | **II** | **III** | **IV** |
|  | **n** | **n** | **n** | **n** | **n** |
| **Evolution** |  |  |  |  |  |
| disease-free | 76 | 204 | 45 | 0 | 0 |
| metastasis | 2 | 20 | 54 | 30 | 7 |
|  |  |  |  |  |  |
| **Tumor location** |  |  |  |  |  |
| Head/neck | 20 | 28 | 18 | 3 | 0 |
| Trunk | 20 | 99 | 25 | 11 | 0 |
| Upper limb | 13 | 27 | 5 | 2 | 0 |
| Lower limb | 18 | 53 | 34 | 11 | 4 |
| Hand/foot | 6 | 13 | 11 | 3 | 1 |
| Others | 1 | 0 | 5 | 0 | 1 |
| Unknown | 0 | 3 | 2 | 0 | 1 |
|  |  |  |  |  |  |
| **Histological Type** |  |  |  |  |  |
| SSM | 22 | 146 | 24 | 9 | 0 |
| NM | 0 | 16 | 42 | 11 | 2 |
| LMM | 19 | 17 | 1 | 0 | 0 |
| ALM | 5 | 8 | 9 | 2 | 1 |
| Others | 1 | 7 | 11 | 0 | 0 |
| Unknown | 31 | 27 | 15 | 8 | 4 |
|  |  |  |  |  |  |
| **Breslow Thickness (mm)** |  |  |  |  |  |
| ≤1.0 | 71 | 159 | 0 | 2 | 0 |
| >1.0-2.0 | 0 | 56 | 14 | 8 | 0 |
| >2.0-4.0 | 0 | 1 | 44 | 5 | 1 |
| >4.0 | 0 | 2 | 29 | 8 | 3 |
| Unknown | 7 | 6 | 12 | 7 | 3 |
|  |  |  |  |  |  |
| **Ulceration** |  |  |  |  |  |
| Yes | 0 | 8 | 44 | 11 | 3 |
| No | 78 | 216 | 55 | 19 | 4 |

^1^Ten samples were excluded due to the lack of relevant information. Abbreviations: m/f, males/females; SSM, Superficial Spreading Melanoma; NM, Nodular Melanoma; LMM, Lentigo Maligna Melanoma; LM, Lentigo Maligna; ALM, Acral Lentiginous Melanoma
