# Supplementary material for: Serum markers improve current prediction of metastasis development in early‐stage melanoma patients: a machine learning‐based study
Source: Mol Oncol. 2020 Jun 24;14(8):1705–18. doi: 10.1002/1878-0261.12732 (PMC7400797; doi:10.1002/1878-0261.12732)
Supplement: Supplementary file 5 — Table S2. Train and test prediction scores in the three domains of variables for the battery of algorithms used. [file MOL2-14-1705-s005.docx]

**Table S2. Train and test prediction scores in the three domains of variables for the battery of algorithms used.**

| **BRESLOW THICKNESS** | | | | | | | | |
| --- | --- | --- | --- | --- | --- | --- | --- | --- |
|  | **Train**  **Balanced accuracy** | **Test**  **Balanced accuracy** | **Train**  **Recall** | **Test**  **Recall** | **Train**  **Precision** | **Test**  **Precision** | **Train**  **Area ROC** | **Test**  **Area ROC** |
| **LR** | 74.92 | 73.02 | 72.65 | 69.50 | 52.98 | 51.69 | 83.45 | 83.72 |
| **SVM** | 74.03 | 73.57 | 65.65 | 64.94 | 56.37 | 58.4 | 82.28 | 81.51 |
| **DT** | 77.93 | 72.56 | 76.81 | 69.06 | 57.07 | 53.5 | 84.07 | 78.72 |
| **NB** | 72.66 | 72.56 | 54.76 | 54.67 | 66.65 | 69.17 | 83.08 | 83.28 |
| **KNN** | 78.69 | 73.26 | 80.32 | 72.94 | 54.99 | 49.67 | 87.65 | 79.17 |
| **CYTOKINES + DCD** | | | | | | | | |
|  | **Train**  **Balanced accuracy** | **Test**  **Balanced accuracy** | **Train**  **Recall** | **Test**  **Recall** | **Train**  **Precision** | **Test**  **Precision** | **Train**  **Area ROC** | **Test**  **Area ROC** |
| **LR** | 65.75 | 59.98 | 62.69 | 52.73 | 35.03 | 29.83 | 71.98 | 65.42 |
| **SVM** | 69.04 | 58.19 | 53.78 | 35.04 | 47.96 | 33.28 | 75.25 | 62.98 |
| **DT** | 73.57 | 57.79 | 70.88 | 47.73 | 46.58 | 30.12 | 78.74 | 58.38 |
| **NB** | 60.62 | 55.52 | 43.01 | 35.40 | 45.60 | 31.44 | 65.9 | 58.50 |
| **KNN** | 72.21 | 56.78 | 74.96 | 52.07 | 39.68 | 26.80 | 79.69 | 56.16 |
| **BRESLOW THICKNESS + CYTOKINES + DCD** | | | | | | | | |
|  | **Train**  **Balanced accuracy** | **Test**  **Balanced accuracy** | **Train**  **Recall** | **Test**  **Recall** | **Train**  **Precision** | **Test**  **Precision** | **Train**  **Area ROC** | **Test**  **Area ROC** |
| **LR** | 82.38 | 80.37 | 79.41 | 77.53 | 59.40 | 57.38 | 90.95 | 89.22 |
| **SVM** | 80.15 | 77.56 | 73.18 | 68.60 | 60.44 | 60.02 | 87.94 | 85.99 |
| **DT** | 86.54 | 76.03 | 87.90 | 70.8 | 62.66 | 53.87 | 92.68 | 85.46 |
| **NB** | 79.73 | 77.79 | 69.09 | 66.02 | 65.68 | 63.46 | 88.45 | 85.62 |
| **KNN** | 85.02 | 74.52 | 86.26 | 70.73 | 58.88 | 46.97 | 92.66 | 81.99 |
